# Metabolism in the Zebrafish Retina

**DOI:** 10.3390/jdb9010010

**Published:** 2021-03-15

**Authors:** Natalia Jaroszynska, Philippa Harding, Mariya Moosajee

**Affiliations:** 1 Institute of Ophthalmology, University College London, London EC1V 9EL, UK; philippa.harding.17@ucl.ac.uk; 2 Moorfields Eye Hospital NHS Foundation Trust, London EC1V 2PD, UK; 3 Great Ormond Street Hospital for Children NHS Foundation Trust, London WC1N 3JH, UK; 4 The Francis Crick Institute, London NW1 1AT, UK

**Keywords:** metabolism, glucose, retina, photoreceptors, RPE, zebrafish, eye development, inherited retinal diseases, retinitis pigmentosa (RP), Leber congenital amaurosis (LCA)

## Abstract

Retinal photoreceptors are amongst the most metabolically active cells in the body, consuming more glucose as a metabolic substrate than even the brain. This ensures that there is sufficient energy to establish and maintain photoreceptor functions during and after their differentiation. Such high dependence on glucose metabolism is conserved across vertebrates, including zebrafish from early larval through to adult retinal stages. As the zebrafish retina develops rapidly, reaching an adult-like structure by 72 hours post fertilisation, zebrafish larvae can be used to study metabolism not only during retinogenesis, but also in functionally mature retinae. The interplay between rod and cone photoreceptors and the neighbouring retinal pigment epithelium (RPE) cells establishes a metabolic ecosystem that provides essential control of their individual functions, overall maintaining healthy vision. The RPE facilitates efficient supply of glucose from the choroidal vasculature to the photoreceptors, which produce metabolic products that in turn fuel RPE metabolism. Many inherited retinal diseases (IRDs) result in photoreceptor degeneration, either directly arising from photoreceptor-specific mutations or secondary to RPE loss, leading to sight loss. Evidence from a number of vertebrate studies suggests that the imbalance of the metabolic ecosystem in the outer retina contributes to metabolic failure and disease pathogenesis. The use of larval zebrafish mutants with disease-specific mutations that mirror those seen in human patients allows us to uncover mechanisms of such dysregulation and disease pathology with progression from embryonic to adult stages, as well as providing a means of testing novel therapeutic approaches.

## 1. Introduction

The zebrafish retina, akin to its human counterpart and the retina of other vertebrates, is one of the most metabolically active tissues in the body [1]. Photoreceptor cells located within the outer layer of the retina account for the vast majority of retinal metabolic activity, and thus represent the epicentre of retinal metabolism [2]. Rods and cones, the two major types of photoreceptors, are responsible for the detection of light and the initiation of phototransduction. Light captured by the photopigment (opsins) in photoreceptors is converted into electrical signals which are passed through bipolar cells to ganglion cells, which form the axons of the optic nerve and carry the stimuli to the visual cortex in the brain [3].

The metabolic profile of the vertebrate retina is unique and complex, encompassing adaptations that enable rod and cone photoreceptors to perform their function and promote survival [4]. Hence, metabolism is key to sustaining visual function throughout life. Photoreceptors in the outer retina and the neighbouring retinal pigment epithelium (RPE) are thought to rely on distinct energy-generating pathways, and the interplay between them contributes to their precise metabolic control [5]. Photoreceptor cells utilise glucose as their primary substrate, but the precise balance between the two leading metabolic pathways, aerobic glycolysis and oxidative phosphorylation (OXPHOS) is yet to be fully elucidated [2,6,7,8]. Numerous inherited retinal diseases (IRDs), in which photoreceptors or RPE cells degenerate, have been investigated using zebrafish mutant models and found to be associated with failure of metabolism [9]. Metabolic dysfunction can arise due to degeneration photoreceptors or RPE cells, failure of substrate delivery or uptake into cells, or the inability of cells to utilise metabolic substrates [4,5,6]. Therefore, the metabolism of individual cell types should be considered in the context of the metabolic ecosystem established by all the cells in the outer retina, because they are highly dependent on each other for sustaining metabolic functions.

The current review will discuss the conserved structure and function of developing and mature zebrafish retina, including evidence from a range of other vertebrate species. We will explore the metabolism and regulatory mechanisms between rods and cones, as well as the RPE and Müller glia cells to sustain visual function. Lastly, the use of zebrafish models of human IRDs will be explored in this context, highlighting how they have contributed to our current understanding of the molecular basis of these disorders and their progression.

## 2. The Zebrafish Retina

### 2.1. Advantages of the Zebrafish as a Model Organism to Study Retinal Metabolism and Disease

**Figure 1:**
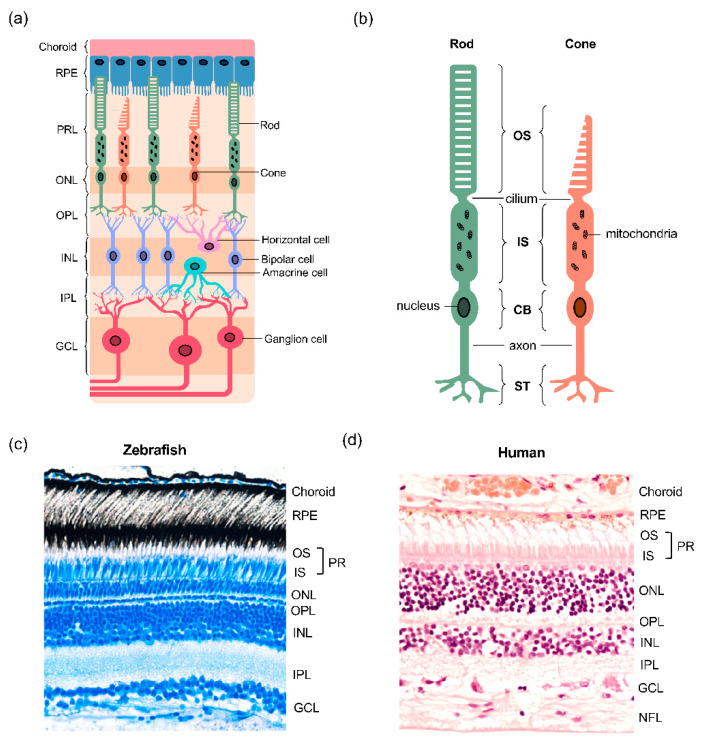
Structural features of the vertebrate retina. (**a**) Overview of the multicellular layered retina. The retinal pigment epithelium (RPE) sits between the photoreceptor layer (PRL) in the outer retina and the choroidal vasculature. The cell bodies of rod and cone photoreceptors are located in the outer nuclear layer (ONL) and form synapses with horizontal and bipolar cells in the outer plexiform layer (OPL). The inner nuclear layer (INL) contains the cell bodies of horizontal, bipolar and amacrine cells, the latter two forming synapses with retinal ganglion cells (RGC) in the inner plexiform layer (IPL); (**b**) structure of the rod and cone photoreceptors. Both photoreceptor types have a light-sensitive outer segment (OS) connected to the inner segment (IS) by a single connecting cilium. The IS is abundant in megamitochondria, an adaptation that maximises energy production. The cell body contains the nucleus and protein synthesis machinery, and the axon separates the cell body (CB) from the synaptic terminals (ST), whilst transmitting the action potential that triggers glutamate neurotransmission release from ST; (**c**,**d**) a comparison of the structure of the human and zebrafish retinae.

The zebrafish (*Danio rerio*) is a well-established model organism, frequently used for investigating the genetic and molecular basis of human development and disease [9,10,11]. In recent years, the use of zebrafish embryos and larvae has proven particularly useful for the study of eye morphogenesis, retinal degeneration and metabolism [12,13,14,15] The cone-rich zebrafish retina makes it possible to isolate these cells for experimental use [16,17], which was previously limited to whole-retina studies due to the challenges in rod-dominated models, such as rodents. Zebrafish embryos develop ex vivo, are transparent, and possess large eyes relative to their body size, making laboratory investigation easier than in mammalian retinae. A crucial benefit of using embryonic zebrafish for ocular research is their fast rate of retinogenesis, reaching adult retina patternation within the first 72 hpf, including a mature laminated structure capable of light detection through established photoreceptors (Figure 1) [18,19,20,21]. The RPE is also differentiated by this time, providing a complete model of the vertebrate retina and its cellular microenvironment. Another key advantage of zebrafish is the ease of genetic manipulation, enabling the production of transgenic fish lines to model monogenic human diseases, such as several inherited retinal diseases (IRDs) [22]. Such genetic amenability coupled with a number of behavioural assays, such as the optokinetic reflex (OKR) and startle response, provide an excellent tool for dissecting disease mechanisms and testing of therapeutic strategies for retinal diseases [23,24,25]. Lastly, a breeding adult zebrafish produces large numbers of embryos, providing large sample numbers for experimental use. 

### 2.2. Conserved Structure of the Zebrafish Retina

The retina is the neural tissue located at the back of the vertebrate eye, responsible for the detection of light and its conversion into electrical signals, which are subsequently sent to the visual centres in the brain, where an image is formed [26]. As in other vertebrates, the mature zebrafish retina consists of three cellular layers, termed outer nuclear layer (ONL), inner nuclear layer (INL) and ganglion cell layer (GCL), and two synaptic layers, termed outer plexiform layer (OPL) and inner plexiform layer (IPL) (Figure 1). The ONL of the retina houses the cell bodies of photoreceptors, the sensory neurons that mediate light-detection and phototransduction (the conversion of light into an action potential) (Figure 1a). Photoreceptor cell bodies (CB) in the ONL contain the nuclei and project axons towards horizontal and bipolar cells, forming synaptic connections in the OPL (Figure 1b). In the zebrafish retina, the photoreceptors are intercalated with the apical projections from the adjacent RPE monolayer (Figure 1c), similar to other vertebrates. In addition, spanning the entire length of the retina are Müller glia (MG), the most common glial cell type in the vertebrate retina.

### 2.3. Zebrafish Photoreceptors

The two major classes of photoreceptors in the retina are rods and cones, whose structure and functions are conserved across vertebrates. Rod photoreceptors are responsible for scotopic vision, occurring in dim-light conditions, whereas cones mediate photopic vision that takes place in the light, as well as colour vision and fine visual acuity. Photoreceptors consist of four distinct subcellular compartments: the outer segment (OS), inner segment (IS), cell body (CB) and synaptic terminals (ST) (Figure 1b). The two major functional segments are the IS and OS, which are linked by a thin connecting cilium that allows for the transport of material between the two compartments [21]. The two segments harbour distinct biochemical reactions that fuel their individual functions. The OS contains photopigment opsins, allowing for the capture of light and subsequent transduction of the signal and the IS is specialised with mitochondria which support the production of energy for energy-dependent transmission of electrical signals [27]. 

While humans possess three types of cone photoreceptors which absorb red, blue and green light, zebrafish possess an additional fourth cone type which absorbs ultraviolet (UV) light and has been linked to zebrafish behaviour, specifically prey capture in daylight and tissue regeneration which are energy-expensive processes [28,29,30,31,32,33]. The different cone types in zebrafish are distinguished by their morphology, defined as either long/short single cones or double cones, as well as by their peak absorption wavelengths based on the single type of opsin they express [34,35,36,37]. Long (L) cones express long wavelength-sensitive (LWS) opsins, more commonly referred to as red-opsins due to their peak sensitivity wavelength of 470 nm. Middle wavelength sensitive cones, M-cones, express medium wave sensitive (MWS) or green opsins (480 nm), while short (S) cones are characterised by their expression of short wavelength-sensitive (SWS1) opsins, which detect blue light [38]. The fourth type of opsin present in the zebrafish photoreceptors is the UV-sensitive opsin, with a maximum wavelength of ~360 nm. 

Individual species differ in their relative ratios of rod and cone photoreceptors and their distributions within the retina, depending on their visual needs. Similar to humans, zebrafish are diurnal, and thus have a high density of cones compared with nocturnal species such as rodents, which are rod dominated [39]. Such high cone density of the zebrafish retina provides a more suitable model for the study of cones and any related pathologies, than mouse models even at embryonic stages of development [40]. The adult zebrafish photoreceptor layer consists of regularly arranged cones surrounded by rods, together forming a mosaic pattern, while human cones are confined to a small area in the central retina called the macula, with surrounding rods occupying the peripheral retina [20,41,42,43]. Thus, humans and fish are comparable in possessing a high density of cones for photopic vision, although their organisation is considerably different. When the photoreceptor fates are specified during embryonic development (from 2dpf), the arrangement of cones appears largely irregular [44]. The crystalline mosaic pattern becomes more apparent as the retina matures and rods differentiate, appearing as a regular lattice in the adult retina, from around 3 months [42,45]. The establishment of the mosaic structure is thought to rely upon the specification of UV cones, as demonstrated by Raymond and colleagues in their study of the *tbx2b* mutant which lacks UV cones and exhibits a dysregulation of the cone column arrangement in the mosaic [17,46,47].

### 2.4. Mitochondria in Zebrafish Photoreceptors

The photoreceptors of zebrafish, and several other teleost species, possess a unique form of mitochondria called megamitochondria which are not present in humans and other mammals [27]. They are significantly larger in size and contain fewer cristae than typical mitochondria that surround them [21]. Tarboush and colleagues studied the ultrastructure of photoreceptor mitochondria in developing and adult zebrafish, revealing dynamic changes in mitochondrial morphology at different developmental stages [48]. They observed striking differences between the morphology of rod and cone mitochondria during early developmental stages, with rods possessing small, electron-dense mitochondria (E-DM), different to the electron-lucid (E-LM), moderately sized mitochondria observed in cones. They further showed that different cone subtypes can be distinguished by the morphology of their mitochondria, suggesting that metabolism is at the forefront of distinct photoreceptor functions. The expansion of mitochondrial subpopulations that give rise to the megamitochondria has been shown to be controlled by ES1, a mitochondrial enlarging factor [49]. Morpholino-mediated knockdown of the *ES1* gene in zebrafish was shown to decrease the size of cone mitochondria, diminishing the number of megamitochondria compared to uninjected embryos. They also found a small reduction in eye size, suggesting that perturbations in metabolism during development may affect fundamental processes such as eye growth [49]. Further studies are required to understand the role of megamitochondria in supporting cone development and functions, as well as the effect of their loss upon eye development as a whole. 

## 3. Metabolism in Photoreceptors

In a functionally mature retina, metabolism is central to visual function, which depends upon numerous energy-dependent reactions requiring adenosine triphosphate (ATP) [50]. The most energy consuming process in neurons is the repolarisation of the plasma membrane after its depolarisation has occurred, to allow the transmission of electrical signals. ATP provides energy for the opening and closing of gated ion channels and maintaining the correct flow of ions [1]. The repolarisation of the plasma membrane is extremely important, as it facilitates the reactivation of the neuron, allowing for transmission of multiple signals. As rod and cone photoreceptors are responsible for the detection and conversion of photons into electrical signals for neurotransmission, generating sufficient energy levels to support these reactions is crucial for allowing the outer retina to respond rapidly to changing light intensity and ensure efficient excitability.

### 3.1. Energy Production in Zebrafish Photoreceptors

Glucose is the major metabolic substrate used by retinal neurons [51]. Glucose flux can be traced in transparent zebrafish larvae through live imaging of the fluorescent glucose analogue 2-NBDG (2-(***N***-(7-Nitrobenz-2-oxa-1,3-diazol-4-yl)Amino)-2-Deoxyglucose), as well as in fixed tissues [52,53,54]. This method has been utilised for pharmacological screens aiming to identify novel antidiabetic agents [53,54]. In retinal disease, similar glucose uptake assays can be applied to screen for therapeutics that may rescue glucose uptake and prevent, or halt, photoreceptor degeneration. Glucose tracing in the retinae of larval and adult zebrafish using 2-NBDG has revealed that all retinal neurons take up some glucose, but the highest proportion gets preferentially taken up by rod and cone photoreceptors, reflecting the findings from the mouse retina [5,55]. 

**Figure 2:**
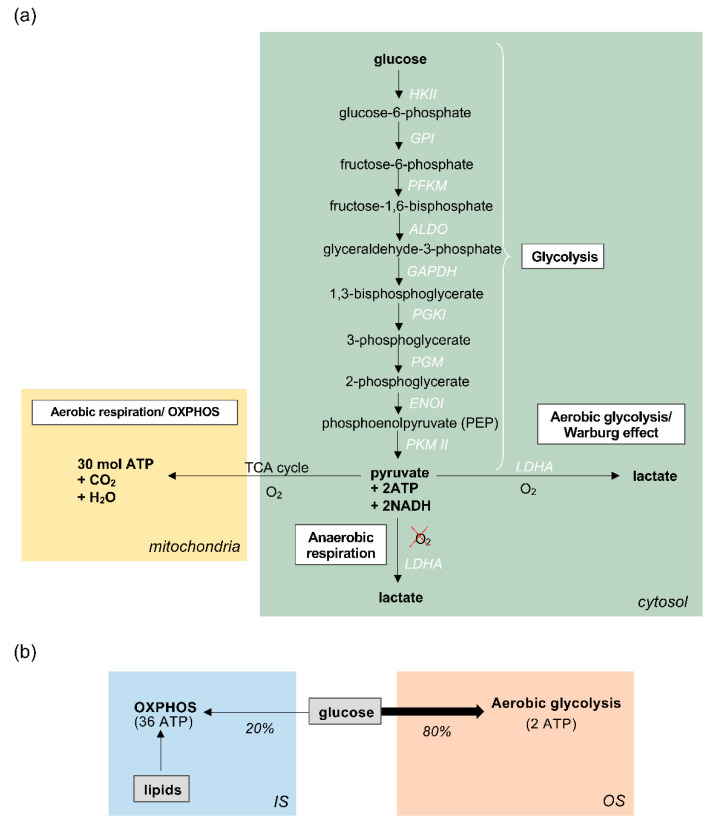
Glucose metabolism in retinal photoreceptors (**a**) Glucose metabolism in photoreceptor cells proceeds first via glycolysis in the cytosol (shown in green), whereby glucose is broken down to produce pyruvate, followed by one of two pathways. In aerobic conditions (oxygen, O_2_) pyruvate is further metabolised via the TCA cycle and oxidative phosphorylation (OXPHOS) in mitochondria. In anaerobic conditions, when O_2_ is absent, pyruvate is converted to lactate by the enzyme Lactate dehydrogenase A (LDHA). In aerobic glycolysis/Warburg effect, the majority of pyruvate is converted into lactate, despite the availability of O_2_ for OXPHOS; (**b**) Summary of the metabolic pathways in photoreceptors. 80% of glucose undergoes aerobic glycolysis in the outer segment (OS), where it produces 2 molecules of ATP for every molecule of glucose. The majority of photoreceptor ATP, approximately 36 molecules per 1 glucose molecule, is generated in the inner segment (IS) by oxidative phosphorylation, although this is likely to be predominantly fuelled by lipids rather than glucose.

Glucose metabolism in the photoreceptors begins with glycolysis in the cytosol, during which a series of enzymatic reactions convert glucose into pyruvate, producing 2 molecules of ATP in the process (Figure 2a). In most cells, the products of glycolysis subsequently fuel oxidative phosphorylation (OXPHOS), also known as the electron transport chain (ETC), within mitochondria. OXPHOS generates approximately 32 mol of ATP for every molecule of glucose, supplying the cell with large amounts of energy. This is known as aerobic respiration, and occurs only in the presence of sufficient oxygen (O_2_) [56]. On the other hand, when oxygen availability is scarce, glycolysis can proceed via an alternative route, known as anaerobic respiration, whereby pyruvate from glycolysis is reduced to lactate, catalysed by the enzyme lactate dehydrogenase A (LDHA). Photoreceptors are unique in that they favour this anaerobic pathway instead of OXPHOS, despite an ample supply of oxygen to the outer retina. When this pathway occurs in the presence of oxygen, it is called aerobic glycolysis, also referred to as the Warburg effect (Figure 2a) [7,8,57,58,59,60]. Interestingly, this metabolic pathway is typically favoured by cells with unusually high metabolic demands, such as cancer cells, whose heightened metabolic activity and large proliferative needs are thought to be supported by this pathway [61,62,63]. 

Strikingly, extensive research into retinal metabolism has revealed that vertebrate photoreceptors have developed an unusual reliance upon aerobic glycolysis, converting over 80% of glucose into lactate via glycolysis, despite the availability of oxygen for OXPHOS [51,64,65,66]. This is vastly different to how other CNS neurons metabolise glucose, which is through the classical oxidative route [67]. However, although it has been speculated that this unusual metabolic adaptation supports the energetically taxing OS reactions that underpin photoreceptor functions, evidence suggests that aerobic glycolysis contributes merely 20% of the total ATP generated by photoreceptors, which is unlikely to be enough to satisfy their large energy requirements [68]. Despite such proclivity for the oxygen-independent glycolytic pathway to metabolise glucose, studies in mammals have revealed that photoreceptors are still responsible for at least 60% of the oxygen consumption in the whole retina. This suggests that photoreceptors do deplete oxygen for OXPHOS, which is therefore likely to be the major source of ATP, but fuel this process by utilising substrates other than glucose [58,59]. A plausible alternative candidate is lipids, which could provide carbons for fuelling the OXPHOS pathway in mitochondria (for a comprehensive review see [68]) and offer an explanation for how photoreceptors maximise ATP synthesis without utilising glucose (Figure 2b). Moreover, the rod and cone ISs house clusters of mitochondria and hence point to a high capacity for OXPHOS [69]. In zebrafish cones, this appears to be particularly enhanced by the presence of megamitochondria, perhaps an evolutionary effort to increase the efficiency of ATP production in cones, which are central to zebrafish vision [30].

Immunohistochemical studies by Calzia and colleagues showed that both larval and adult zebrafish rods express respiratory complexes I and IV, and ATP synthase in their OSs, as demonstrated through their co-localisation with the rod OS marker zpr3 [70]. This suggests that there may be an additional source of ATP in zebrafish rods in the form of extramitochondrial OXPHOS, in contrast to the dependence of cones on mitochondrial catabolism [70]. Although their results confirm the expression of the metabolic enzymes in question in rods, it remains inconclusive whether this is a rod-specific adaptation, or a general zebrafish photoreceptor feature. 

### 3.2. The role of Aerobic Glycolysis in Photoreceptors

Several studies, including some in zebrafish, have explored why photoreceptors have evolved to convert glucose into lactate through aerobic glycolysis, rather than using OXPHOS where the ATP yield would be significantly higher [63,71,72]. It has been speculated that this is an evolutionary attempt to overcome the absence of mitochondria in the OS [73]. However, it is becoming increasingly clear that aerobic glycolysis may be implemented by photoreceptors not for catabolic, energy-generating purposes, but rather to facilitate anabolic, energy-requiring functions. Anabolic metabolism includes the biosynthesis of relevant cellular material from smaller building blocks, usually intermediates derived from a metabolic pathway. Although photoreceptors are terminally differentiated, they require daily regeneration of their light-sensitive OS and thus exhibit a heightened need for biosynthesis. This is due to the daily shedding of approximately 10% of their OS mass and its phagocytosis by RPE cells [74,75]. This is a regenerative mechanism that maintains sufficient levels of visual pigments in the OS for successful light detection and phototransduction. Hence, it would appear that photoreceptors favour aerobic glycolysis in order to satisfy such anabolic requirements and supply metabolic intermediates for use as building blocks OS biosynthesis [8,76,77]. Another study showed that upon stimulation with light, there is a decrease in pyruvate production due to the phosphorylation and inhibition of the enzyme PKM2, thereby causing a build-up of glycolytic intermediates which may take part in anabolic reactions and provide the biomass for OS synthesis [78,79]. Others have also speculated that aerobic glycolysis is more prevalent in proliferating cells during development or in cancer, although further studies in zebrafish could help us understand the dynamics of metabolism from larval stages through to adulthood [80,81].

### 3.3. Metabolic Changes in Light and Darkness

The primary role of vertebrate photoreceptors is to capture light and pass on visual information to other retinal neurons for processing. Rods and cones have different light sensitivities which allows them to appropriately respond to both bright and dim light. In the dark, the requirement for ATP and its rate of synthesis is higher. OXPHOS generates approximately 84% of the total energy in the dark, an increase of 23% compared to its energy contribution in the light [1,66,82,83]. Most of the reactions occurring during darkness take place in the IS, where ATP is required to repolarise the membrane to maintain the ‘dark current’ [83,84]. In brief, in the absence of light, cGMP in the OS keeps Na^+^/Ca^2+^- K^+^ channels open, causing a continuous influx of Na^+^ and Ca^2+^ ions into the cell, resulting in the depolarisation of the membrane. This change in membrane potential opens voltage gated Ca^2+^ channels near the synaptic terminals, thereby allowing for these ions to enter the cell and trigger neurotransmitter release into the synaptic cleft. In order to maintain the dark current, and counteract the accumulation of intracellular Na^+^, photoreceptors focus their energy on repolarising the membrane. This is mediated by the Na^+^/K^+^-ATPase, located in the mitochondrion-rich photoreceptor IS which requires energy from ATP to actively export Na^+^ ions against their concentration gradient [85,86]. The phototransduction pathway is conserved across vertebrates which has been demonstrated in a number of studies in zebrafish [35,87,88,89,90]. Maintaining this electrochemical gradient has a high bioenergetic demand, hence OXPHOS is favoured, and this activity is reflected by increased oxygen (O_2_) consumption in the IS in dark conditions versus in the light, as demonstrated in rodent studies [59,91]. More studies are required in zebrafish to understand oxygen consumption, as well as mitochondrial and glycolytic metabolism in light and dark, as well as in healthy fish compared to those with IRDs. Approaches such as the Seahorse analysis, which can be used to assay both OXPHOS and glycolysis in zebrafish larvae, could be beneficial in furthering our understanding of any zebrafish-specific metabolic adaptations [92,93], and may in part explain the presence of megamitochondria and how they relate to photoreceptor development and zebrafish vision, both in health and disease.

In the light, the functions of photoreceptors shift, as do their metabolic demands. The bioenergetic focus of photoreceptors in these conditions is driving phototransduction, which is initiated in the light-sensitive OS compartment. In the diurnal zebrafish, this energy requirement may be more elevated to support their robust visual functions in the daytime, as discussed above. The membrane disks that constitute the OS contain an abundance of opsins in cones and rhodopsin in rods, which are members of the G protein coupled receptor (GPCR) family [94]. These molecules capture light, thereby initiating a signal transduction cascade that modulates neurotransmitter release at the synapses and ensure that visual information is relayed onto second-order retinal neurons and ultimately the brain [94]. First, light captured by opsins or rhodopsin, activate the associated G protein transducin which in turn drives pde6c-mediated hydrolysis of cGMP. This causes its dissociation from the cGMP-gated Na^+^/Ca^2+^ channels, thereby closing them and preventing further influx of ions. Importantly, this means that in response to light, the cell hyperpolarises, which diminishes the requirement for the active transport of Na^+^ ions out of the cell through the Na^+^/K^+^-ATPase pump. ATP consumption by these pumps is therefore significantly reduced in the light, as is the need for OXPHOS. 

All neurons must maintain the correct intracellular concentrations of ions such as Ca^2+^, in order to drive the depolarization of their membranes. Mitochondria are of particular importance in the regulation of intracellular Ca^2+^ levels in photoreceptors, providing necessary control of key processes such as phototransduction, neurotransmission and metabolism [95,96]. Giarmarco et al. showed that zebrafish mitochondria are involved in the compartmentalization of distinct Ca^2+^ pools required for phototransduction in the OS, and neurotransmission at the synaptic termini, by forming mitochondrial clusters that serve as a barrier between the different pools [96]. Additionally, mitochondria in the cell body take up Ca^2+^ from the cytosol via the mitochondrial Ca^2+^ uniporter (MCU), driving ATP production through OXPHOS by stimulating dehydrogenases in the TCA cycle [97]. Knockouts of MCU in zebrafish and mice showed mild alterations in metabolism, while phototransduction and neurotransmission remained unperturbed [98]. Interestingly, the absence of MCU did not completely halt Ca^2+^ uptake into the mitochondria in these KOs, suggestive of an alternative route for its uptake [98]. This is a possible adaptation to maximise Ca^2+^ homeostasis and mitochondrial metabolism, and thereby sustain key photoreceptor functions. Another study aimed to overexpress MCU in zebrafish to drive the uptake of Ca^2+^ to pathological levels, which surprisingly only caused mild mitochondrial disruptions, with no photoreceptor degeneration, or impact on phototransduction [95]. This further suggests that photoreceptor mitochondria are essential to maintain Ca^2+^ homeostasis, and thereby protect the invaluable metabolic pathways that drive photoreceptor functions. In addition, significant alterations in Ca^2+^ levels may be a cause or consequence of mitochondrial, and therefore metabolic dysfunction and contribute to photoreceptor degeneration in IRDs [89,96,99,100,101].

## 4. Metabolism in the Retinal Pigmented Epithelium (RPE) and Müller Glia

Being amongst the most energy-consuming cells in the body, photoreceptors require large quantities of nutrients to fulfil their high metabolic demand. The delivery and uptake of metabolic substrates and the removal of the metabolic products and waste relies upon support from the RPE [4]. The RPE is a monolayer of highly polarised cells that separate the outer retina from the choroidal blood supply and ensure that sufficient amounts of glucose reach rods and cones [102,103,104,105]. Aside from sustaining the photoreceptors’ metabolic functions, RPE cells themselves require energy to many functions in the retina and withstand the high levels of reactive oxygen species and light-induced damage to which they are exposed daily, impacting metabolism [106,107,108]. A number of studies in mice and in vitro models have highlighted the differences between photoreceptor and RPE cell metabolism [5,108,109]. 

Before reaching the retina, glucose from the choroid travels through the RPE via GLUT-1 transporters located on both the basolateral and apical RPE surfaces [110,111,112,113,114]. However, despite possessing an abundance of glucose channels and thus an ample supply of a potential substrate, conditional GLUT-1 knockouts in mice have shown disturbances in catabolic and anabolic metabolism in the photoreceptors and Müller glia, but not the RPE cells. This suggests that RPE cells rely upon alternative substrates to generate energy [115]. Kanow et al. have shown that in return for not using up the glucose from the choroid, RPE cells receive a large supply of lactate secreted from photoreceptors, specifically the glycolytic pathway. Utilising lactate as a substrate allows them to generate ATP and satisfy their own metabolic needs through OXPHOS or reductive carboxylation [5,116]. They also pointed to the ability of lactate to suppress glycolysis in RPE cells using in vitro models, a positive feedback loop presumed to occur in order to prevent glucose from being consumed by the RPE, maximising its supply to the photoreceptors [5]. Zebrafish *Glut-1*, (also known as *Slc2a1*) knockdowns have previously been generated [117,118], however these have been used to study blood-brain barrier development, and little is known on the effects of the deletion in the developing and aging retina. Lastly, in addition to reduced glucose metabolism, RPE cells also consume nitrogen metabolites such as ammonia, nucleotides and amino acids differently to photoreceptors [111], consistent with the notion that the two cell types have distinct but interdependent energetic profiles that contribute to a metabolic ecosystem. Interestingly, the dysfunction of RPE mitochondria has been associated with the degeneration of not only the RPE itself, but also retinal photoreceptors, and may play a role in IRDs [109].

Müller Glia (MG) are the dominant glial cell type in the vertebrate retina, with an established role in supporting retinal neurons in structural and functional aspects, as well as assisting with the unique regenerative capabilities of zebrafish [119,120,121,122]. Their morphology and functions have been extensively studied in both larval and adult zebrafish, although our understanding of their metabolism remains limited [121,123]. Therefore, most information regarding their metabolic profiles currently comes from mammalian studies, and whether there are any zebrafish-specific adaptations that would be of relevance to metabolism in the outer retina is yet to be explored in detail.

Similar to the RPE, MG are thought to be adapted to maximise the photoreceptor metabolic capacity by tailoring their own metabolic profiles to complement and enhance that of photoreceptors, as reflected in the different metabolic enzyme expression and ability to modulate their metabolism as part of their supportive functions [124,125]. Interestingly, their metabolism is unlike that of the RPE, and is in fact more parallel to photoreceptors, relying largely on glycolysis. Indeed, MG express GLUT-1, and alongside photoreceptors have been shown to be the primary entry point of glucose into the rodent retina [55]. Glucose metabolism in MG produces large amounts of lactate which has been speculated to feed the photoreceptors [126,127]. They also supply of a range of metabolites to photoreceptors and are essential for nitrogen metabolism [128,129]. However, the role of MG in metabolism has predominantly been studied in the context of retinal ganglion cell metabolism, and further studies on the relationship between photoreceptor and MG in zebrafish are necessary to fully understand the metabolic ecosystem in the outer retina.

## 5. Metabolic Imbalance and Retinal Degeneration

Dysfunction and metabolic failure can result in inherited retinal diseases (IRDs), such as Leber congenital amaurosis (LCA), retinitis pigmentosa (RP) and rod/cone dystrophies [130,131,132,133,134,135,136]. Presently around 261 genes have been associated with IRDs, most with orthologues in zebrafish [135,136]. Key functional pathways such as phototransduction [137], inter-compartment transport of cellular material through the connecting cilium [138,139], OS biosynthesis [68], retinoid metabolism [140,141,142], and supportive cellular processes in RPE and choroid can be disrupted by genetic mutations, and once decompensated, can trigger apoptosis, which ultimately leads to progressive loss of vision. Zebrafish models of IRDs have been investigated to further our understanding of the effect of disease on retinal metabolism.

### 5.1. Retinitis Pigmentosa (RP)

**Figure 3:**
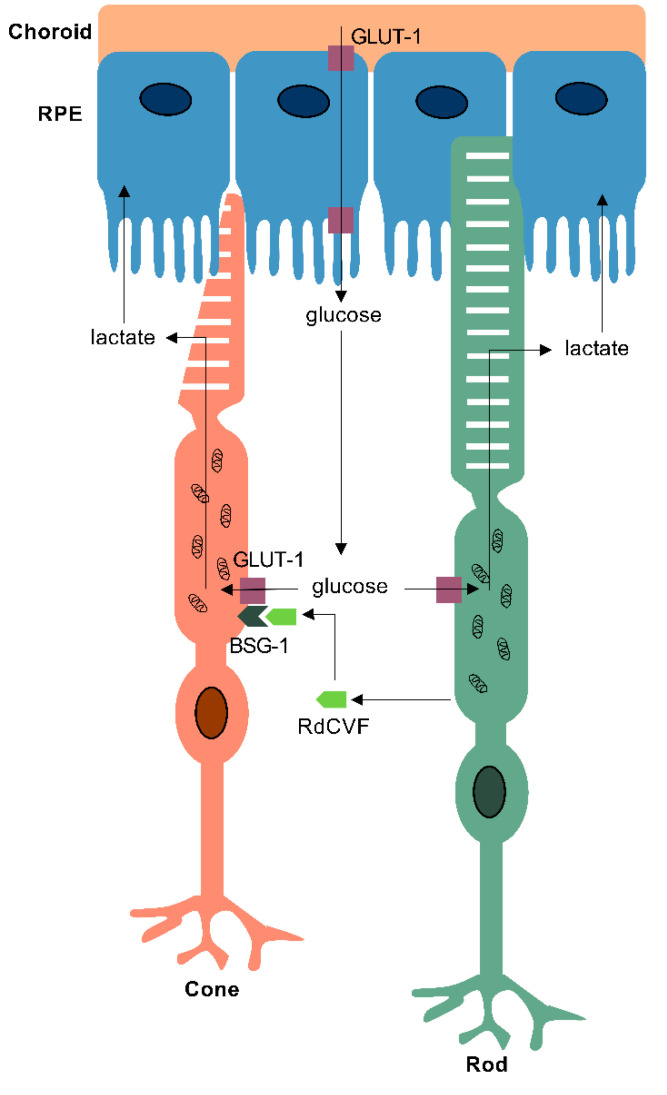
The metabolic ecosystem in the outer retina. The distinct metabolic pathways operating in rod and cone photoreceptors and retinal pigmented epithelium (RPE) cells work together to establish a precisely balanced metabolic ecosystem. The RPE mediates the transepithelial transport of glucose from the choroidal vasculature to rod and cone photoreceptors via GLUT-1 transporters, to fuel aerobic glycolysis in photoreceptor outer segments. Rods release rod-derived cone viability factor (RdCVF) which enhances glucose uptake into cones, by forming a complex with its transmembrane receptor, basigin-1 (BSG-1) and GLUT-1. Both rods and cones produce lactate as an end-product of their metabolism, which is subsequently secreted and taken up by RPE cells, which utilise it as their primary metabolic substrate. Thus, the precise metabolic relationships between the three cell types are crucial for sustaining the functions of the outer retina, and their imbalance may result in degeneration.

RP affects 1 in 4000 individuals, and is the most common cause of blindness amongst working age adults in the UK, with over 80 causative genes [143,144]. It is characterised by the primary loss of rod photoreceptors leading to nyctalopia and peripheral visual field loss. This is followed by secondary non-cell autonomous death of cones, which results in the loss of central vision and complete blindness. The observation that cone death triggers the degeneration of cones led to a number of investigations into the possible role of rods in promoting cone survival [145,146,147,148]. Studies in chick and mouse models revealed that vertebrate rods release a thioredoxin-like protein, Rod-derived Cone Viability Factor (RdCVF) encoded by the *nxnl1* gene, which acts upon cones to enhance their absorption of glucose [146,149]. The discovery of the cone receptor for RdCVF, Basigin-1 (BSG-1), helped elucidate the molecular mechanism by which RdCVF supports cones in a healthy retina. Evidence from in vitro and in vivo studies in mice revealed that RdCVF forms a complex with its receptor, BSG-1, and the GLUT-1 transporter also expressed on the surface of cones, enhancing glucose uptake into cones (Figure 3) [150,151]. Therefore, as a consequence of rod death caused by one of the heterogenous genetic mutations, the release of RdCVF ceases, thereby leading to an impairment in glucose uptake into cones. A reduction in glucose uptake means that there is an insufficient supply of the key metabolic substrate for aerobic glycolysis in the OS of cones, without which cones are unable to meet their metabolic demands [152]. Insufficient anabolism results in impaired OS regeneration, shown in patients presenting with shortening of photoreceptor OS, leading to cellular dysfunction and eventually apoptotic cell death [153]. Zebrafish possess orthologues of the *nxnl1*, *bsg* and *GLUT-1* genes, and protective effects of rods upon cones have been demonstrated, suggesting that this mechanism is also conserved in the zebrafish [145]. Most recent therapeutic approaches for IRDs aim to rescue cones and prevent severe vision loss and blindness through the replacement of the trophic factor RdCVF by gene therapy to enhance the uptake of glucose via GLUT-1 [154,155]. 

However, although zebrafish are known to express RdCVF, to what extent their cones depend on rod-derived factors remains elusive. The *ebony* and *ivory* zebrafish models of photoreceptor degeneration have revealed a non-cell autonomous degeneration of rods and cones, consistent with the presence of a diffusible factor such as RdCVF [156]. However, others have speculated that zebrafish may lack the same rod-cone interactions observed in mammals. For example, Morris and colleagues used a transgenic zebrafish line and immunohistochemistry to demonstrate that driving rod-specific degeneration does not necessarily result in secondary death of cones in all zebrafish models of retinal disease [157]. Given the low proportion of rods within the zebrafish retina, it seems probable that the expression of RdCVF would be lower than in mammals and insufficient in supporting cone metabolism alone, thereby driving evolutionary efforts to otherwise maximise energy production. An example of this could be the megamitochondria in cones, or additional OXPHOS capacity in the OSs, which would allow cones to increase energy metabolism without relying on rods. Further studies in zebrafish will be necessary to confirm how comparable photoreceptor metabolism is in different vertebrate species, particularly to assess the suitability of individual zebrafish models of IRDs to model human disease.

### 5.2. Leber Congenital Amaurosis (LCA)

LCA represents the most severe type of IRD, with patients often presenting with blindness in the first year of life. There are 26 causative genes that affect a number of different cellular processes, leading to the disease phenotype [158]. One such gene is *KCNJ13*, encoding the inward rectifying potassium channel Kir7.1, expressed in both the photoreceptors and the RPE [159]. A recent study examined the retinal pathophysiology of LCA in a zebrafish model, the *obelix^td15^* (*obe^td15^*) mutant, which carries a missense mutation in *kcnj13* (c.502T>Cp.[Phe168Leu]) and exhibits an ocular phenotype similar to that of LCA patients, albeit with a later onset [160]. By 12 months, histological and OCT studies in *obe^td15^* mutants revealed a general thinning of the retina and severe photoreceptor and RPE degeneration, accompanied by decreased visual acuity, as determined by OKR. Analysis of retinal ultrastructure in the mutant fish showed mitochondrial alterations in cone photoreceptors, including their enlargement and a more compact distribution within the IS at 12 months. Mitochondria are able to withstand stress conditions, such as changing Ca^2+^ concentrations, to a degree before employing compensatory mechanisms, for example changing their size or shape [95,160]. Such reliance and adaptability may explain the delayed onset of photoreceptor degeneration in the *obe^td15^* model [160]. In the RPE, mitochondrial number and size increased progressively in the mutant fish in contrast to WTs, which exhibited a reduction in mitochondrial size with age, confirming that mitochondrial alterations are a pathological sign of metabolic dysfunction. Further, they found that the clearance of phagosomes was compromised in comparison to WT, indicative of RPE dysfunction and failure to degrade OSs shed by photoreceptors [160]. Failure of phagosomal clearance has been implicated in oxidative stress, accumulation of lipids and mitochondrial dysfunction. They further demonstrated evidence of metabolic dysfunction in the mutant fish using Seahorse analysis, which showed a reduction in the oxygen consumption rate and ATP production compared to WTs, suggestive of a metabolic impairment. These organelle changes preceding photoreceptor degeneration highlight the importance of metabolism in retinal and RPE pathologies, making energy metabolism an attractive therapeutic target for future IRD treatments.

## 6. Conclusions

It is becoming increasingly clear that metabolism is fundamental to photoreceptor function and survival in all vertebrates. The highly conserved, carefully controlled metabolic pathways enable rod and cone photoreceptors in the outer retina to maintain distinct catabolic and anabolic reactions and differentially respond to changes in light intensity, mediating visual function. The interplay between photoreceptors and the surrounding cells ensures that their respective metabolic demands are met, by supplying the correct substrates to fuel their independent metabolic reactions. Thus, disturbing metabolism in photoreceptor cells, RPE and/or the choroid, can result in retinal degeneration and consequent sight loss. Due to their diurnal nature and vision-dependent behavioural features, such as prey capture, zebrafish appear to be particularly adapted to maximise energy metabolism in cones, although further studies are necessary to obtain a more complete picture of their metabolic profiles and how it compares to other vertebrate species. Additionally, understanding how glucose metabolism influences the development, survival, and viability of rods and cones in the zebrafish retina can provide crucial insight into the possible mechanisms behind human retinal disease pathologies, since retinal structure and function are highly conserved across all vertebrates. This is key to identifying a common metabolic pathway to target, which would aid the development of therapeutic approaches for a range of IRDs and prevent photoreceptor degeneration irrespective of the specific genetic causes, which remain heterogenous and difficult to tackle individually.
